# The combination of etoposide and platinum for the treatment of thymic neuroendocrine neoplasms: A retrospective analysis

**DOI:** 10.1002/cam4.6245

**Published:** 2023-06-23

**Authors:** Yelan Guan, Qifeng Yao, Yue Hao, Xiaohong Zeng, Wenxian Wang, Xiadong Gu, Jing Xiang, Yan Sun, Zhengbo Song

**Affiliations:** ^1^ Department of Phase I Clinical Trial Zhejiang Cancer Hospital, Hangzhou Institute of Medicine (HIM), Chinese Academy of Sciences Hangzhou Zhejiang China; ^2^ Postgraduate training base Alliance of Wenzhou Medical University Zhejiang Cancer Hospital Hangzhou Zhejiang China; ^3^ Department of Thoracic Oncology Surgery Zhejiang Cancer Hospital, Hangzhou Institute of Medicine (HIM), Chinese Academy of Sciences Hangzhou Zhejiang China; ^4^ The Second Clinical Medical College of Zhejiang Chinese Medical University Hangzhou Zhejiang China; ^5^ Department of Thoracic Medical Oncology Zhejiang Cancer Hospital, Hangzhou Institute of Medicine (HIM), Chinese Academy of Sciences Hangzhou Zhejiang China

**Keywords:** efficacy, etoposide, platinum, safety, thymic neuroendocrine neoplasms

## Abstract

**Background:**

To provide real‐world outcomes for the combination of etoposide and platinum as a first‐line treatment for advanced thymic neuroendocrine neoplasms (TNENs).

**Methods:**

Retrospective analysis was performed on patients with advanced TNENs confirmed by pathology who received etoposide combined with platinum as a first‐line chemotherapy in our institution between 2010 and 2022.

**Results:**

A total of 16 patients were included in this study. Twelve patients (75%) received etoposide combined with cisplatin, and four patients (25%) received etoposide combined with carboplatin. Efficacy was evaluated in all patients, with an objective response rate of 31.3%. One patient achieved a complete response, four achieved a partial response, and in eight patients the disease remained stable; the disease control rate was 81.3%. The median progression‐free survival (PFS) was 7.2 months with a 95% confidence interval (CI) of 2.1–12.3 months. The median overall survival (OS) was 50.4 months with a 95% CI of 32.1–68.8 months. No significant difference in efficacy was observed between the treatment groups with regards to PFS (*p* = 0.095) and OS (*p* = 0.061). Treatment‐related adverse events were observed in all 12 patients when evaluated for toxicity, manifesting as hematologic toxicity. Grade 3–4 bone marrow suppression occurred in six patients (50%). No treatment‐related deaths were recorded.

**Conclusion:**

This retrospective analysis, conducted in a real‐life setting, suggests that the combination of etoposide and platinum has a promising anti‐tumor activity in advanced TNENs, with a clinically significant overall response rate.

## 1 BACKGROUND

Thymic neuroendocrine neoplasms (TNENs) are extremely rare mediastinal tumors that originated from neuroendocrine cells. Rosai and Higa were the first to reported this type of tumor in 1972.^1^ This disease has been reported to account for <5% of all anterior mediastinal tumors.^2^ The estimated incidence of TNENs in the United States is 0.18 per 1 million people.^3^, ^4^ According to the 2021 World Health Organization (WHO) classification of thymus tumors,^5^ TNENs involve carcinoid/neuroendocrine tumors, including typical carcinoid (TC), atypical carcinoid (AC), small cell carcinoma (SCC), and large cell neuroendocrine carcinoma (LCNEC). Histologically, TNENs are classified into low‐grade, intermediate grade and high‐grade, corresponding to well differentiated, moderately differentiated, and poorly differentiated tumors, respectively. Furthermore, the degree of malignancy increases with the grade. In a manner that differs from neuroendocrine tumors in the lungs, TNENs are more likely to be associated with endocrinopathies (e.g., Cushing syndrome), multiple endocrine neoplasia‐1 (MEN‐1), and ectopic adrenocorticotropic hormone syndrome (EAS).^6^, ^7^, ^8^

Although most patients with this type of disease present with distant metastases at the time of presentation, TNENs appear to fare better than neuroendocrine tumors of pulmonary origin in terms of survival. TNEN has long been considered a mediastinal neuroendocrine carcinoma of the lungs, because this condition is pathologically consistent with an origin in the lungs. According to the literature, the overall 5‐year survival of this condition varies from 31% to 52.8%.^9^, ^10^, ^11^ Based on the unassailable first‐line status of etoposide in combination with platinum for the treatment of advanced pulmonary neuroendocrine tumors, clinicians empirically prefer the combination of etoposide and platinum for the treatment of advanced TNENs. However, due to the rarity of this disease, there is no evidence from randomized controlled studies to confirm the efficacy of etoposide in combination with platinum‐based treatment of this disease.

Based on this background, our current research provided a key data relating to the efficacy and safety of etoposide plus platinum as a first‐line treatment for advanced TNENs in a real‐world setting.

## 2 METHODS

### 2.1 Study design and patients

We retrospectively recruited patients who had been diagnosed with TNENs at the Zhejiang Cancer Hospital between 2010 and 2022, and received etoposide in combination with platinum at advanced stages of disease. We extracted a range of information from electronic medical records, including basic patient information, pathology type, histological grade, chemotherapy regimen, efficacy, and toxicity. The enrollment criteria for this study were as follows: (1) patients with pathologically confirmed TNENs (carcinoid, small cell carcinoma, and large cell neuroendocrine cancers); (2) patients with TNENs staged IVA or IVB according to the American Joint Commission on Cancer (AJCC) staging 8th edition Tumor‐Node‐Metastasis (TNM) staging or Masaoka‐Koga staging principles (pleural or pericardium dissemination or lymphatic/hematogenous metastasis); (3) received at least 2 cycles of first‐line chemotherapy involving etoposide plus platinum, with evaluable tumor lesions; (4) etoposide combined with platinum as an adjuvant chemotherapy regimen was considered as first‐line treatment if distant metastasis occurred during or within 6 months after the end of the last treatment. The exclusion criteria were as follows: (1) pathologically confirmed compound small cell carcinoma of thymic origin; (2) two or more primary site tumors, and (3) active disease requiring hormonal or hospital treatment.

### 2.2 Procedures

All patients in our study were treated according to the National Comprehensive Cancer Network (NCCN) guidelines for small cell lung cancer (SCLC). Etoposide plus cisplatin (EP) or carboplatin (EC) was used as first‐line chemotherapy in all advanced patients until the patient developed intolerable toxicity or received up to 4 to 6 cycles. The dosage of etoposide was 100–120 mg/m^2^ on day 1 to 3, and the dosage of cisplatin was 60–75 mg/m^2^ on day 1, q21d. Patients were treated with an EC regimen using etoposide 100 mg/m^2^ on days 1 to 3 with carboplatin (area under the curve of 5–6), q21d. All patients were evaluated for post‐treatment efficacy by enhanced computed tomography (CT) of the chest and abdomen, and magnetic resonance imaging (MRI) of the brain to measure the size of the lesions. Toxicity assessment was performed according to the Common Terminology Criteria for Adverse Events (CTCAE version 5.0).

### 2.3 Outcomes

All patients included in this study had received at least two circle treatments of etoposide in combination with platinum and had measurable lesions. Efficacy was assessed according to the version 1.1 of the Response Evaluation Criteria in Solid Tumors (RECIST) for patients who had received 2 cycles of treatment. Overall survival (OS) was defined as the time between the diagnosis of advanced disease and the time the patient died or last follow‐up. Progression‐free survival (PFS) was defined as the time between receiving first‐line chemotherapy and disease progression or last follow‐up. Efficacy was classified as a complete response (CR), a partial response (PR), stable disease (SD), and progress disease (PD). Objective response rate (ORR) was defined as the proportion of patients achieving CR and PR out of all evaluable patients. Disease control rate (DCR) was defined as the proportion of patients receiving CR, PR, and SD out of all patients evaluable for efficacy.

### 2.4 Statistical analysis

Data were analyzed by SPSS version 25.0 for Windows (SPSS Inc.). The median PFS and OS time were estimated by the Kaplan–Meier method. The test results of this study were reported using two‐sided *p*‐values, and *p*‐values <0.05 were statistically different. All patients in this single sample study were included in the statistical analysis. The follow‐up rate was 100% and the median follow‐up time was 59.8 (95% confidence interval [CI], 59.2–60.3) months. The last follow‐up visit was February 24, 2023.

## 3 RESULTS

### 3.1 Patient characteristics

Between the January 1, 2010 and the February 24, 2022, 16 patients were enrolled from our institution (Zhejiang Cancer Hospital). All patients were eligible. None of the patients had a concurrent MEN‐1 or other disease of the endocrine system. However, two patients had a combination of superior vena cava syndrome, presenting with facial swelling, hoarseness, and shortness of breath. The baseline characteristics of the patients are shown in Table 1.

**TABLE 1:** The characteristics of the 16 patients.

Characteristics, *n* (%)	Total (*n* = 16)
Age, median(range), year	53 (13–77)
Sex, male/female	12/4 (75.0 vs. 25.0)
ECOG PS	
1	14 (87.5)
2	2 (12.5)
Smoking status	
Never	7 (43.8)
Former or current	9 (56.3)
Tumor size	
<5.7 cm	7 (43.8)
≥5.7 cm	7 (43.8)
Not available	2 (12.5)
Histologic classification	
Atypical carcinoid	3 (18.8)
Poorly differentiation NEC	11 (68.8)
Highly differentiated NEC	2 (12.5)
Chemotherapy regimens	
Etoposide + carboplatin	4 (25.0)
Etoposide + cisplatin	12 (75.0)
Surgical procedures	
Yes	6 (37.5)
No	10 (62.5)
Radiotherapy	
Yes	10 (62.5)
No	6 (37.5)
Number of distant metastatic organ	
1	6 (37.5)
≥2	10 (62.5)

Abbreviations: ECOG, eastern cooperative oncology group; NEC, neuroendocrine carcinoma; PS, performance status.

The median age at diagnosis was 53 years (range: 13–77 years). All patients were of Chinese Han ethnicity, 12 were male and four were female; the male‐to‐female ratio was also 3:1. Most patients had an Eastern Cooperative Oncology Group (ECOG) performance status score of 1 (87.5%) and two patients (12.5%) had a score of 2. Seven patients (43.8%) had never smoked, and nine patients (56.3%) had a history of previous smoking. The pathological type of most patients in this study was neuroendocrine carcinoma (NEC). Half of the patients (*n* = 11, 68.8%) showed a poorly differentiated histological type, two cases (12.5%) involved atypical carcinoid, and three cases (18.8%) involved well differentiated neuroendocrine carcinoma.

In addition, the median tumor size was 5.8 cm (range: 3.5–20.0). The ki‐67 index were available for 12 patients and the median ki‐67 index was 70% (range: 5%–95%). All patients received a first‐line chemotherapy regimen of etoposide in combination with platinum. Twelve patients (75%) received chemotherapy with EP and four patients (25%) treated with EC. Six patients underwent surgical excision, including partial (*n* = 2) and complete excision (*n* = 4). Of them, five (31.3%) patients had undergone surgery prior to treatment, and one patient with mediastinal lymph node metastasis progressed after 2 cycles of EP neoadjuvant chemotherapy and underwent surgical excision. Ten (62.5%) patients received radiation therapy during treatment. More than half of the patients (10/16, 62.5%) had at least two distal organ metastases, and six patients (37.5%) had no more than two distant metastases. Furthermore, the most common distant metastatic organ was the lung or pleura, accounting for 68.8% of all patients. The other common metastases were liver (8/16, 50%), bone (6/16, 37.5%), adrenal glands (*n* = 3, 18.8%), brain (*n* = 1, 6.3%), and pancreas (*n* = 1, 6.3%).

### 3.2 Efficacy

**FIGURE 1:**
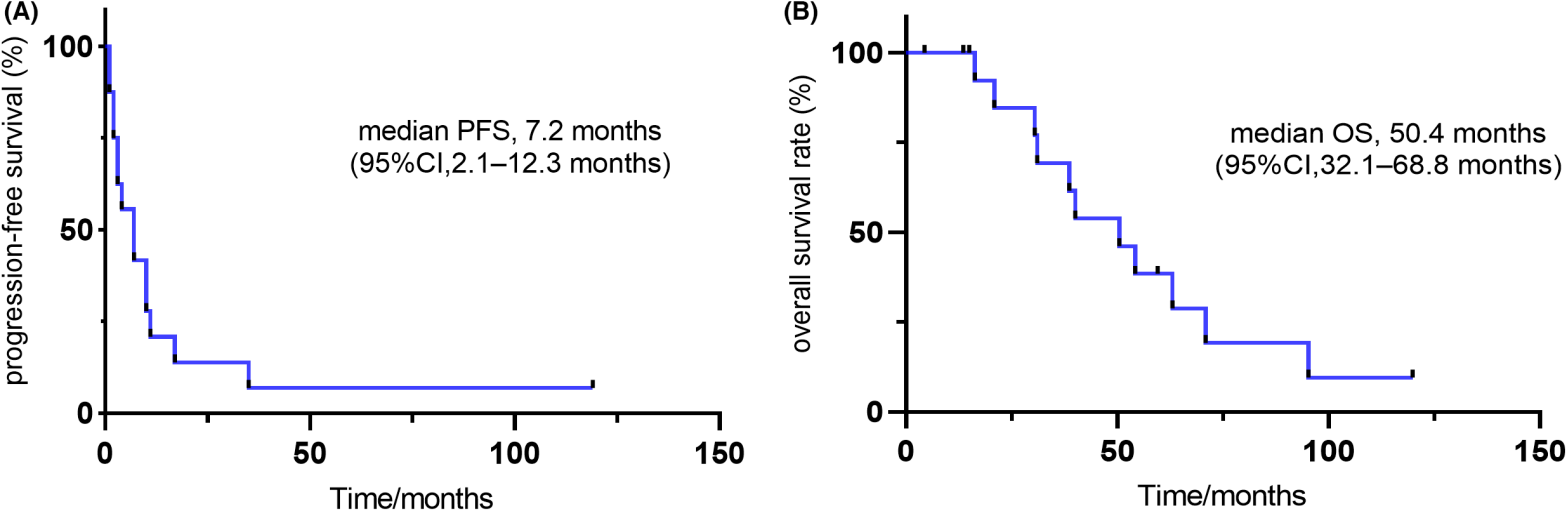
Survival curve of 16 patients. (A) progression‐free survival (PFS), (B) overall survival (OS).

**FIGURE 2:**
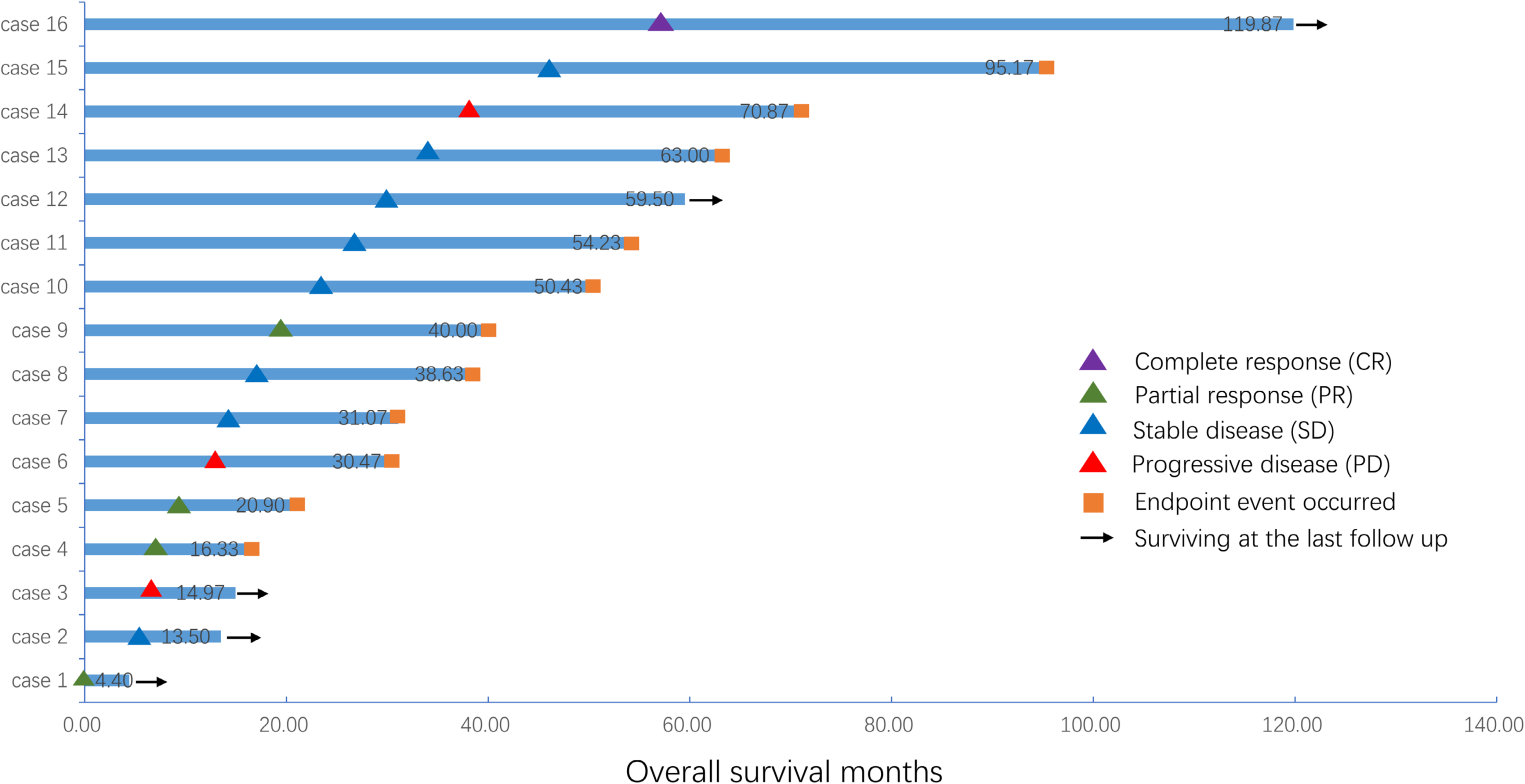
The swimmer plots of etoposide plus platinum in first‐line treatment of advanced thymic neuroendocrine tumors (TNETs).

The observed therapeutic effects are shown in Table 2. The median follow‐up time was 59.8 (95% CI, 59.2–60.3) months. Outcomes were evaluable for all patients. One patient achieved a complete response. PR and SD were evaluated in four (25.0%) and eight (50.0%) patients, respectively. Moreover, three patients (18.8%) experienced disease progression after 2–3 cycles of chemotherapy with EP regime; the pathological types were AC, SCC, and LCNEC, respectively. Overall, the assessed objective response rate and disease control rate were 31.3% and 81.3%, respectively. For 16 patients, the median PFS (mPFS) was 7.2 months (95% CI, 2.1–12.3 months), and the median OS (mOS) was 50.4 months (95% CI, 32.1–68.8 months) (Figure 1). In thesubgroup analysis, the mPFS for patients with AC (*n* = 3) and NEC were 7.2 months (95% CI, 1.2–13.1 months) and 17.4 months (95% CI, 0.0–42.8 months), respectively (*p* = 0.463). The mOS for patients with NEC and AC were 40.0 months (95% CI, 19.1–60.9 months) and 95.0 months (95% CI, not available), respectively (*p* = 0.187). However, most inevitably develop disease progression or drug resistance. Swimming plots of etoposide plus platinum for the first‐line treatment of advanced TNENs are shown in Figure 2. By the follow‐up cut‐off time, only two patients had stable disease and were followed regularly in the outpatient clinic. Most patients (*n* = 14, 87.5%) will inevitably develop disease progression or resistance. Finally, 11 patients (68.8%) had died and five patients (31.3%) remained alive at the end of the study.

**TABLE 2:** The efficacy of etoposide plus platinum in the first‐line treatment of advanced TNENs.

Efficacy evaluation index	Total (*n* = 16)
Complete response	1 (6.3%)
Partial response	4 (25.0%)
Stable disease	8 (50.0%)
Progressive disease	3 (18.8%)
Objective response rate	31.3%
Disease control rate	81.3%
Median progression‐free survival (95%CI)	7.2 (2.1–12.3) months
Median overall survival (95%CI)	50.4 (32.1–68.8) months

Abbreviation: CI, confidence interval.

There was no significant difference in efficacy between the treatment groups in terms of PFS (*p* = 0.095) and OS (*p* = 0.061). Notably, one patient presented with only right supraclavicular lymph node metastasis (TNM staging N2, IVb) with a pathological type of poorly differentiated NEC; this patient achieved complete remission after 6 cycles of chemotherapy with the EP regimen. The patient did not receive radiotherapy or surgery in addition to chemotherapy. At the end of follow‐up, the patient was still alive with a PFS of 119 months and an OS of nearly 10 years. In our research, 62% of patients survived more than 3 years, and 29% survived for more than 5 years. For patients who progressed after treatment with first‐line etoposide combined with platinum, the following strategies were applied: irinotecan combined with paclitaxel, temozolomide + capecitabine, irinotecan monotherapy, gemcitabine combined with oxaliplatin or docetaxel, anti‐angiogenesis targeted therapy (anlotinib or apatinib), and re–challenge treatment with first‐line chemotherapy regimens re‐challenge.

In this study, six patients received surgery and ten patients received radiation therapy. Of these, three cases developed distant metastases after adjuvant radiotherapy after radical excision. Figure S1 depicts OS analysis for the surgery and radiotherapy subgroups. As expected, there were no differences in outcomes between the surgical (*p* = 0.403) and radiotherapy subgroups (*p* = 0.277). The median OS for patients receiving surgery resection or not receiving surgery resection was 63.0 (95%CI, 43.0–83.0) months and 38.6 (95%CI, 16.5–60.7) months, respectively. In the radiotherapy subgroup, the median OS for patients receiving radiotherapy or not receiving radiotherapy was 38.6 months (95%CI, 11.8–65.4) and 54.2 (95%CI, 23.7–84.8) months, respectively. Univariate COX regression analysis associated with PFS and OS is shown in Table S1. No significant *p*‐values were generated in our study, and no risk factors were identified by multivariate analysis.

### 3.3 Toxicity

For all 12 evaluable patients, treatment‐related adverse events manifested as hematological toxicity. Grade 1–2 myelosuppression was identified in six patients (50%); three cases were in the EC group and three were in the EP group. The remaining six patients (50%) developed grade 3–4 myelosuppression. In addition, three patients had grade IV myelosuppression and two patients had grade III myelosuppression, respectively. Of concern, all patients with grade 3 and above hematologic toxicity were in the EP treatment group. However, after symptomatic management of all 12 patients, myelosuppression improved, and the original treatment regimen was continued or the treatment dose was reduced. No patient experienced treatment‐related death.

## 4 DISCUSSION

According to previous reports, TNENs accounts for 0.4% of all neuroendocrine tumors.^12^, ^13^, ^14^, ^15^ The extreme rarity of TNENs has led to a lack of prospective clinical trials and large, high‐quality cohort studies. Only a few retrospective studies have investigated the prognosis of this disease.^3^, ^6^, ^16^ Standard guidelines or expert consensus has not been established for the treatment of TNENs. For almost four decades, countless new drugs have been tested as standard treatments for SCLC; however, all have failed. EP or EC regimens have become the first‐line standard of care for lung‐derived neuroendocrine tumors. The chemotherapy strategy of etoposide combined with platinum has also been widely used in clinical practice for the treatment of TNENs derived from neuroendocrine cells. However, there are no retrospective studies of the efficacy of such regimens on TNENs. To the best of our knowledge, this research is the first real‐world description to date of the effects of etoposide in combination with platinum treatment in patients with advanced TNENs.

In fact, there is considerable controversy relating to the full management of thymic tumors, including the tumor staging system, the timing of surgery, the safety of postoperative adjuvant radiotherapy, the appropriate population for adjuvant chemotherapy, and the standard of care for advanced patients. With regards to the characteristics of the patients, our study was similar to those previously reported, with a median age at diagnosis of 53 years; this was similar to previous study that reported with a median age of diagnosis of 54 years, and a similar male to female ratio (3:1).^4^ The efficacy of etoposide plus platinum in TNENs has not been described previously. Earlier studies have referred to the 2015 WHO classification of tumors of the thymus,^17^ distinguishing between thymoma and other subtypes of malignancies classified as subtype C; that is, thymic carcinomas (TCs). Table 3 provides a review of literature from previous studies on the efficacy of etoposide alone or in combination with other drugs in the treatment of advanced TCs.

**TABLE 3:** The studies on etoposide alone or combined with other drugs in the treatment of thymic carcinomas.

Authors	Year	Study	Regimen	N	CR	Stage^a^	PR	ORR	mPFS, mo	mOS, mo
Loehrer et al.^18^	2001	Clinical trial	VIP	8 TC	0	III–IV	2	25.0%	NA	approximate 27.5
Kawasaki et al.^19^	2014	Retrospective	CODE	7 TC	0	III–IV	5	71.4%	NA	NA
Bluthgen et al.^20^	2016	Retrospective	Etoposide	15 TC	0	II–IV	2	13.3%	4.0	13.0
Current study	2023	Retrospective	EP/EC	16 TNENs	1	IV	4	31.3%	7.2	50.4

Abbreviations: CODE, cisplatin, vincristine, doxorubicin, and etoposide; CR, complete response; EC, etoposide and carboplatin; EP, etoposide and cisplatin; mo, months; mOS, median overall survival; mPFS, median progression‐free survival; NA, not available; ORR, objective response rate; PR, partial response; VIP, etoposide, ifosfamide, and cisplatin.

^a^ Masaoka Stage.

A previous clinical trial^18^ explored the efficacy of etoposide plus ifosfamide and cisplatin in eight patients with advanced TCs. Two patients achieved a PR, with an ORR of 25%. Similarly, this study did not elaborate on the pathological subtypes of TCs. Another study^19^ from Japan included seven TCs with Masaoka stages III–IV who underwent surgical resection after induction chemotherapy and were administered with cisplatin, vincristine, doxorubicin, and etoposide (CODE) regimen. In the best evaluation of the efficacy of chemotherapy, five patients observed PR and two patients had SD; six patients underwent R0 resection.

In addition, a retrospective study^20^ was conducted on the efficacy of oral etoposide in 15 patients with stage of II–IV thymic carcinomas. Most patients (55%) received at least two other regimens prior to oral etoposide. Two patients with advanced TC who were treated with oral etoposide monotherapy achieved a PR with an ORR of 13.3%, a mPFS of 4.0 months and a mOS of 13.0 months, respectively. However, this particular study did not indicate the subtype of TCs. Furthermore, the most recent case^21^ reported a patient with advanced thymic SCC with liver metastases, bilateral pleural effusion and pericardial fluid in which all evaluable lesions disappeared after 4 cycles of EC regimen chemotherapy. Six months later, CT scan revealed recurrence of the primary tumor. After 1 cycle rechallenged chemotherapy, surgical resection was performed and the tumor did not recur after 2.5 years of follow‐up.

Considering these studies, significant tumor remission was observed in patients with unresectable locally advanced or advanced TCs, either with etoposide alone or in combination with other cytotoxic agents. However, previous studies did not specifically classify pathological subtypes of TCs. With an in‐depth understanding of the biology of thymic tumors, the subtypes of thymic carcinomas continue to be refined, and the treatment strategies for different prognostic subtypes of tumors are bound to differ. The above‐mentioned case report showed the better anti‐tumor activity for the EC regimen in advanced TNENs. Our study is the first to describe the efficacy of etoposide in combination with platinum in advanced TNENs, reporting data on ORR of 31.3%.

TNENs are biologically different from neuroendocrine tumors of pulmonary origin, less aggressive than SCLC, and have a lower median age of diagnosis. Furthermore, these patients are less likely to develop brain metastases, not associated with smoking, and have a longer survival compared to SCLC.^4^, ^6^ The 5‐year survival rate for advanced TNENs observed in our study was 29%. Surgical treatment of R0 in resection is considered the primary treatment option for TCs. However, for TNENs, this is not certain. In our study, five patients who progressed during or within 6 months of postoperative adjuvant chemoradiotherapy. The reason for the failure of adjuvant chemoradiotherapy is all due to distant metastases. Since our study included patients who were at an advanced stage of diagnosis or those who had failed adjuvant radiotherapy after surgery, the value of surgery resection and radiotherapy was not applicable. However, as shown in Figure S1, increasing the sample size may have resulted in separation of the two curves for the two groups. Further exploration of the value of surgery and radiotherapy for TNENs is expected in future. In addition, the outcomes of induction chemotherapy with EC or EP regimens for the treatment of advanced TNENs also need to be confirmed.

Multi‐center, data from prospective study may warrant additional attention to support the position of etoposide plus platinum for the first‐line treatment of advanced TNENs. Remarkably, immune checkpoint inhibitors (ICIs) have shown surprising anti‐tumor activity for extensive‐stage SCLC and the efficacy of ICIs in advanced TNENs is also worth investigating. Since thymic tumor cell lines are extremely rare, research in the experimental field also requires the technical dissemination of researchers to expand research and treatment options for this rare tumor.

Unfortunately, the univariate COX regression analysis performed in this study did not yield significant survival‐related risk factors. This may be related to our study design and limited sample size. In addition, our research has several limitations that need to be considered. First, this study was a retrospective study carried out in a single center; thus, retrospective defects, such as recall bias and selection bias, may have existed. Second, this research included three patients with atypical carcinoid; thus, our study population was not exclusive to patients with neuroendocrine carcinoma. The prognosis between carcinoid tumors and neuroendocrine carcinoma may differ. Third, the limited sample size of this study may have influenced our conclusion.

## 5 CONCLUSION

This retrospective analysis, conducted in a real‐life setting, suggests that etoposide a combination with platinum has promising anti‐tumor activity for the treatment of advanced TNENs, with a clinically significant overall response rate.

## AUTHOR CONTRIBUTIONS

**Yelan Guan:** Conceptualization (equal); data curation (equal); formal analysis (equal); methodology (equal); software (equal); writing – original draft (equal). **Qifeng Yao:** Conceptualization (equal); data curation (equal); formal analysis (equal); methodology (equal); writing – original draft (equal). **Yue Hao:** Software (equal); writing – review and editing (equal). **Xiaohong Zeng:** Software (equal); writing – review and editing (equal). **Wenxian Wang:** Data curation (equal). **Xiadong Gu:** Data curation (equal). **Jing Xiang:** Data curation (equal). **Yan Sun:** Supervision (equal). **Zhengbo Song:** Conceptualization (lead); methodology (lead); supervision (lead); writing – review and editing (lead).

## FUNDING INFORMATION

None.

## CONFLICT OF INTEREST STATEMENT

The authors have no relevant interests to disclose.

## ETHICS APPROVAL AND CONSENT TO PARTICIPATE

The study design was approved by the Ethics Committee of Zhejiang Cancer Hospital with the approval number IRB‐2022‐62. The Ethics Committee of Zhejiang Cancer Hospital approved the waiver of written informed consent for retrospective study.

## Supporting information


Figure S1

Table S1

## Data Availability

The datasets used and/or analyzed in the current study are available from the corresponding authors on reasonable request.
