# Supplementary figures and images for: The combination of etoposide and platinum for the treatment of thymic neuroendocrine neoplasms: A retrospective analysis
Source: Cancer Med. 2023 Jun 23;12(15):16011–8. doi: 10.1002/cam4.6245 (PMC10469660; doi:10.1002/cam4.6245)

**A**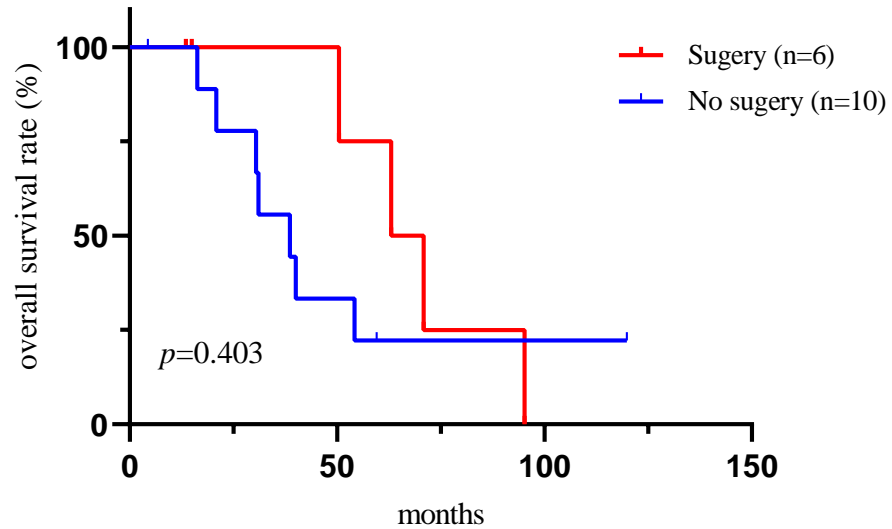**B**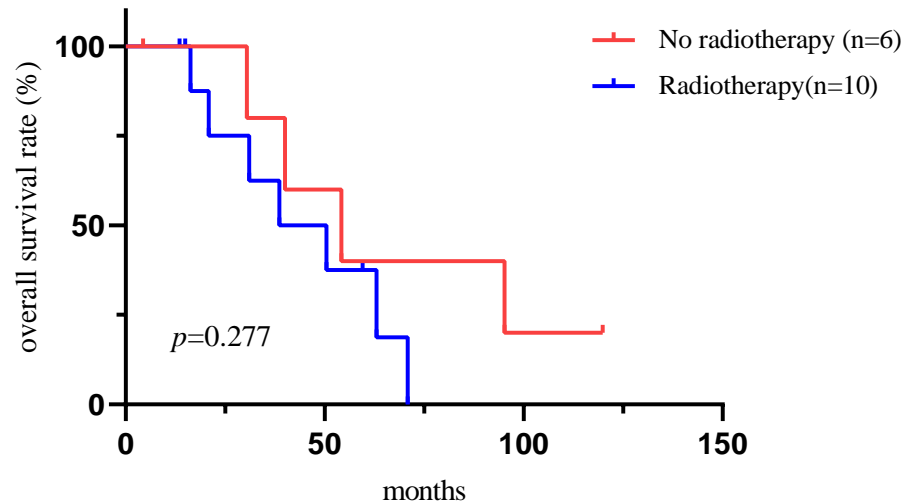

Supplement: Supplementary file 1 — Figure S1 [file CAM4-12-16011-s002.pdf]
