# Supplementary material for: The combination of etoposide and platinum for the treatment of thymic neuroendocrine neoplasms: A retrospective analysis
Source: Cancer Med. 2023 Jun 23;12(15):16011–8. doi: 10.1002/cam4.6245 (PMC10469660; doi:10.1002/cam4.6245)
Supplement: Supplementary file 2 — Table S1 [file CAM4-12-16011-s001.docx]

**Supplementary** **Table 1.** The univariate cox regression analysis of PFS and OS

|  | **Univariate of PFS** | | **Univariate of OS** | |
| --- | --- | --- | --- | --- |
|  | P value | HR 95% CI | P value | HR 95% CI |
| Year of diagnosis(<2010 vs ≥2010) | 0.778 | 0.859(0.298–2.479) | 0.101 | 3.312(0.793–13.829) |
| Gender (Male vs Female) | 0.523 | 0.656(0.180–2.390) | 0.575 | 0.639(0.133–3.061) |
| Age (<60 vs ≥60) | 0.658 | 0.765(0.234–2.501) | 0.718 | 1.261(0.359–4.430) |
| Smoking (Never vs current/former) | 0.872 | 0.915(0.312–2.689) | 0.744 | 1.229(0.355–4.253) |
| PS score (1 vs 2) | 0.387 | 2.032(0.408–10.118) | 0.250 | 2.724(0.494–15.029) |
| Tumor size (<5.7 cm vs ≥5.7cm) | 0.099 | 0.326(0.086–1.235) | 0.068 | 0.142(0.018–1.151) |
| Histological grade (non-low differentiation vs low differentiation) | 0.941 | 1.044(0.338–3.227) | 0.244 | 2.237(0.577–8.668) |
| Surgery therapy (No vs Yes) | 0.194 | 2.078(0.689–6.267) | 0.408 | 0.584(0.163–2.086) |
| Radiotherapy (No vs Yes) | 0.451 | 1.580(0.481–5.187) | 0.287 | 2.103(0.535–8.265) |
| Number of metastases (1 vs ≥2) | 0.311 | 1.855(0.561–6.134) | 0.392 | 1.982(0.414–9.478) |
| Liver metastasis (No vs Yes) | 0.239 | 2.076(0.615–7.006) | 0.389 | 1.763(0.485–6.413) |
| Lung metastasis (No vs Yes) | 0.312 | 1.787(0.580–5.508) | 0.763 | 1.232(0.318–4.776) |
| Bone metastasis (No vs Yes) | 0.918 | 0.939(0.284–3.106) | 0.353 | 0.556(0.162–1.1917) |
| PS, performance status; PFS, free-progression survival; OS, overall survival | | | | |
